# Validation of standard operating procedures in a multicenter retrospective study to identify -omics biomarkers for chronic low back pain

**DOI:** 10.1371/journal.pone.0176372

**Published:** 2017-05-01

**Authors:** Concetta Dagostino, Manuela De Gregori, Christian Gieger, Judith Manz, Ivan Gudelj, Gordan Lauc, Laura Divizia, Wei Wang, Moira Sim, Iain K. Pemberton, Jane MacDougall, Frances Williams, Jan Van Zundert, Dragan Primorac, Yurii Aulchenko, Leonardo Kapural, Massimo Allegri, on behalf of the PainOmics Group

**Affiliations:** 1 Anesthesia, Critical Care and Pain Medicine Unit, Division of Surgical Sciences, Department of Medicine and Surgery, University of Parma, Parma, Italy; 2 PainTherapy Service, Fondazione IRCCS Policlinico San Matteo, Pavia, Italy; 3 Institute of Epidemiology II, Research Unit of Molecular Epidemiology, Helmholtz Zentrum München, German Research Center for Environmental Health, Neuherberg, Germany; 4 Genos, Glycoscience Research Laboratory, Zagreb, Croatia; 5 Laboratory of Biochemistry and Genetics, Division of Pneumology, Department of Molecular Medicine, Fondazione IRCCS Policlinico San Matteo, Pavia, Italy; 6 School of Medical and Health Sciences, Edith Cowan University, Joondalup, Western Australia, Australia; 7 IP Research Consulting SASU (PHOTEOMIX) - Rex de Chaussée, Noisy le Grand, France; 8 Department of Twin Research and Genetic Epidemiology, King’s College London, London, United Kingdom; 9 Department of Anesthesiology, Critical Care and Multidisciplinary Pain Center, Ziekenhuis Oost-Limburg, Genk, Belgium; 10 St. Catherine Specialty Hospital, Zabok, Croatia; 11 Eberly College of Science, The Pennsylvania State University, University Park, Pennsylvania, United States of America; 12 University of Split School of Medicine, Split, Croatia; 13 University of Osijek School of Medicine, Osijek, Croatia; 14 Children's Hospital Srebrnjak, Zagreb, Croatia; 15 PolyOmica, Groningen, The Netherlands; 16 Carolinas Pain Institute, Winston-Salem, North Carolina, United States of America; University of Hong Kong, HONG KONG

## Abstract

Chronic low back pain (CLBP) is one of the most common medical conditions, ranking as the greatest contributor to global disability and accounting for huge societal costs based on the Global Burden of Disease 2010 study.

Large genetic and -omics studies provide a promising avenue for the screening, development and validation of biomarkers useful for personalized diagnosis and treatment (precision medicine). Multicentre studies are needed for such an effort, and a standardized and homogeneous approach is vital for recruitment of large numbers of participants among different centres (clinical and laboratories) to obtain robust and reproducible results. To date, no validated standard operating procedures (SOPs) for genetic/-omics studies in chronic pain have been developed.

In this study, we validated an SOP model that will be used in the multicentre (5 centres) retrospective “PainOmics” study, funded by the European Community in the 7th Framework Programme, which aims to develop new biomarkers for CLBP through three different -omics approaches: genomics, glycomics and activomics.

The SOPs describe the specific procedures for (*1*) blood collection, (*2*) sample processing and storage, (*3*) shipping details and (*4*) cross-check testing and validation before assays that all the centres involved in the study have to follow.

Multivariate analysis revealed the absolute specificity and homogeneity of the samples collected by the five centres for all genetics, glycomics and activomics analyses.

The SOPs used in our multicenter study have been validated. Hence, they could represent an innovative tool for the correct management and collection of reliable samples in other large-omics-based multicenter studies.

## Introduction

Chronic low back pain (CLBP) is broadly defined as pain, muscle tension, or stiffness localized below the costal margin and above the inferior gluteal folds, with or without sciatica, which persists for 12 weeks or more [1]. The prevalence of chronic low back pain (CLBP) has been rising with a variable prevalence in the USA up to 10.2% [2] and in Brazil up to 9.6% [3]. It accounts for considerable direct and indirect burdens for society, industry and healthcare systems [4, 5] representing the fifth most common reason for medical treatment, costing up to 50 billion dollars in the United States annually [6].

Low back pain (LBP) encompasses a group of mixed pain syndromes with poor correlation between clinical manifestations and variable underlying mechanisms, anatomical lesions, neural pathways (neuropathic and nociceptive) and molecular pathologies. The high inter-individual variability in pain sensation and analgesic with similar “macroscopic” pathology (e.g. disc herniation, arthrosis) is not well understood [7]. Various psychosocial characteristics influence symptoms and prognosis in LBP and these include demographic factors (educational status, age, gender), occupational factors (employment), mental health morbidity (anxiety, depression), perception of pain and disability (pain intensity and expectation of persistent pain) and other psychological factors (fear avoidance, catastrophising, illness perceptions) [8].

To add to the heterogeneity of low back pain syndromes, given there is an underlying inflammatory process involved in the experience of pain, our work in other diseases with inflammatory components [9] suggests that biomarkers have a potential role to play in predicting the risk of disease development and prognosis. The majority of studies that have investigated genetic biomarkers have focused their attention predominantly on disk degeneration and not so much on the low back pain syndrome [10, 11].

Under the 7th Framework Programme The European Community has funded a project called “Multi-dimensional -omics approach to stratification of patients with low back pain” (Acronym: PainOmics). As part of this study we will perform a retrospective study to compare the “omics biomarkers” in approximately 4000 patients with CLBP to those of about 8000 control individuals without CLBP with three different types of -omics: genomics, glycomics (analysis of specific changes in glycan structures) and activomics (analysis of enzymatic activity of numerous post-translational modification proteins).

Our aim is to find biomarkers associated with chronic pain and with the response to specific treatments, in order to obtain the safest and the most effective management of CLBP for each patient.

As these studies require large sample sizes in order to find statistically significant results, enrolment is usually done in various clinical centres. Moreover, the analysis of biological material is also usually done in geographically separated laboratories. Consequently, harmonisation of the procedures used in collecting, handling (e.g. length of time to processing, storage, centrifugation, etc.), shipping and, finally, analysing the samples is of prime importance in order to keep variance to a minimum. Unfortunately, until now there are no standard operating procedures (SOP) for sample collection, validated for use in multicentre chronic pain studies.

Hence, SOPs were developed to provide details for conducting the study, phase by phase, with written instructions to achieve uniformity of the procedures used for obtaining patient blood for -omics analysis techniques, storing the samples, and shipping the aliquots to the specialized laboratories.

In this study we present the results of the validation of these SOPs, by showing their efficacy in all the three specialized laboratories for the “omics” analysis included in the “PainOmics” Consortium. We used the samples collected from the first 10 patients enrolled in each of the 5 Clinical Centres to validate the specific procedures hat least in triplicate test. In fact, the variations in blood collection between several Centres is a common problem leading to misleading results in many–omics studies. The reproducibility of the SOPs cannot be assumed but requires empirical testing, and this is the very point of this pilot study.

## Materials and methods

We validated SOPs used in the PainOmics retrospective study to find -omics biomarker to stratify patients with chronic low back pain recruited in the second middle of 2014. The Institution that granted permission is the Ethics Committee of each Center where patients were enrolled after signed informed consentand it was registered on http://clinicaltrials.gov (NCT02037789).

In particular, this study is approved by Ethic Committee of:

- University of Parma (UNIPR)—Italy;
- Fondazione IRCCS Policlinico San Matteo Hospital (OSM)—Italy;
- St. Catherine Specialty (St-Cat)—Croatia;
- Ziekenhuis Oost-Limburg Autonomeh Verzorgingsinstelling (ZOL)—Belgium;
- The Center for Clinical Research (CPI)—USA;
- Edith Cowan University (ECU)—Australia.

and the results of this study were already published in our manuscript on BMJ Open [12].

The SOPs were validated on 10 patients enrolled by each of the clinical centres for the PainOmics retrospective study. In the Table 1 we list the centres participating in the study and highlight their role. The SOPs were discussed before the beginning of the trial and then finally adopted and presented to the Ethics Committee of each clinical centre. We developed three SOPs; DNA SOP, Plasma SOP, and Serum SOP for genomics, glycomics and activomics analyses, respectively. All the clinical centres had to guarantee that the biological samples from each patient are prepared, stored, and shipped following the analytical procedures defined in the each of three SOPs.

**Table 1:** Participating centres of the PainOmics study.

Name of the Centre	Activity	Sample Code
University of Parma (UNIPR)–Italy	Enrolment of patients	UPRRT
Fondazione IRCCS Policlinico San Matteo Hospital (OSM)—Italy	Enrolment of patients	OSMRT
St. Catherine Specialty Hospital (St-Cat)—Croatia	Enrolment of patients	STCAT
ZiekenhuisOost-Limburg Autonome Verzorgingsinstelling (ZOL)—Belgium	Enrolment of patients	ZOLRT
The Center for Clinical Research (CPI)—USA	Enrolment of patients	CPIRT
Genos DOO Za Vjestacenje i analizu (GENOS DOO)—Croatia	Glycomics analysis	
Helmholtz Zentrum Muenchen Deutsches Forschungszentrum FuerGesundheit Und UmweltGmbh (HMGU)—Germany	Genomics analysis	
IP Research Consulting SASU(PHOTEOMIX)—France	Activomics analysis	
Edith Cowan University (ECU)—Australia	Enrolment of patients	
King’s College London (KCL)—UK	Enrolment of patients	
PolyOmica—The Netherlands	Statistical analysis	

All the patients undergo blood sampling for -omics determinations. The samples are then divided into two vacutainers containing EDTA for genetic and glycomic analyses and into one serum vacutainer with clot activator plus gel for activomics analyses.

Each sample is coded as follow:

PO—RT—acronym of participating centre—progressive number of enrolment–GEN/GLY/ACT respectively for genomics, glycomics and activomics analyses.

“PO” indicates the acronym of Pain OMICS; “RT” indicates the retrospective study.

Each centre will keep the list of codes assigned to its patients, at least until the end of the study.

Within the SOPs a specific time line was fixed, in order to standardize the maximum time frame used for every single step during the process. A detailed method description with defined guidelines was created for the following steps: collection of patient blood sample by using printed labels on each cryotube; transfer of samples to the laboratory; processing, aliquoting and freezing of each sample; also shipping conditions were described including time and storage requests.

The amount of DNA and concentration, as well as numbers of required aliquots required for glycomics and activomics were defined and deviations from SOPs are allowed only if properly justified and shared with the laboratory supervisor (S1–S3 Files).

Finally, a procedure was set up to manage clinical samples whereby all samples are validated for sample integrity (i.e. frozen, bar coded) and entered into a database for subsequent analyses. The database identifies the storage location, temperature, number of freeze-thaw cycles, remaining volume, and details on multiple aliquoting analyses. The primary database is linked via barcode references to a second database that identifies screening results for the multiple markers to be tested. The database also collates sample data from the same patient in order to manage data collected sequentially over the study. The data will be centralized in a specific web-based database (REDCap^®^).

### Validation of the SOPs

A cross-check between the central laboratory and each laboratory of the clinical centres is a key step before performing the analyses. Therefore, the three specialized -omics laboratories described above, received samples from the first 10 patients enrolled by each of the five clinical centres in order to validate the procedures.

#### DNA SOP

The quality and concentration of DNA samples extracted from blood need to be validated to check if they were sufficient for downstream analysis. We identified a number of key technical aspects that contribute to inter-assay variability. These include simple steps such as using different DNA extraction kits (e.g., magnetic beads, columns, etc.).

DNA was extracted from whole blood and subsequently stored at -20°C or -80°C. DNA samples were shipped on dry ice to the central laboratory. Of the first ten enrolled patients from each clinical centre, 2 μg of DNA with a concentration of at least 50 ng/μL was used for validation (S1 File). DNA degradation, DNA integrity as well as sex determination of DNA samples were analysed in all test samples. The DNA quality was assessed by gel electrophoresis and spectrophotometric quantification. The DNA quality was defined according to the ratio of absorbance (nm) at 260/280 (not less than 1.8) and at 260/230 (between 1.8 and 2.2). For sex determination of DNA samples an amelogenin polymerase chain reaction (PCR) was performed. The length of the gene amelogenin is different depending on the X or Y chromosome and thus can be used to distinguish male and female samples. Moreover, this method evaluates if the DNA is suitable for further processing.

High quality DNA samples are required to generate genome-wide genotype data using high-throughput Illumina and Affymetrix genotyping platforms.

#### Plasma SOP

The blood dedicated to glycomics analysis was mixed well in the collection tube for balanced clotting. It was left at room temperature before centrifugation, which should be performed within 1 h. After separation of the plasma, the specimen was promptly divided into aliquots of 1 mL using well-sealed freezing containers and immediately stored at -80°C or -20°C. All aliquots that have not been taken for local use were shipped on dry ice to the central laboratory for collective measurement. The aliquots must be kept at each laboratory to use for cross-check testing to be done at the time of the assays by the central laboratory (S2 File). To enable this highly sophisticated and novel analysis of glycomics at Genos ltd. in Croatia, a custom-built instrument that will combine nano-LC from Waters and an ESI-TOF detector from Bruker was procured and installed. Upon initial installation a full test of the validation procedure was completed. In particular, glycomics analyses were performed immunoglobulin G (IgG) isolated from plasma samples by affinity chromatography using 96-well monolithic plates with Protein G as previously described [13]. N-glycans were released from total proteins and IgG by overnight deglycosylation with N-glycosidase F (PNG-ase F). Released N-glycans were fluorescently labelled with a 2-aminobenzamide (2-AB) fluorescent tag and purified by hydrophilic interaction liquid chromatography (HILIC) solid phase extraction (SPE). The labelled N-glycans were analysed by hydrophilic interaction chromatography on a Waters Acquity UPLC instrument using a Waters BEH Glycan chromatography column, 100 mM ammonium formate, pH 4.4, as solvent A and acetonitrile as solvent B. The system was calibrated using an external standard of hydrolyzed and 2-AB labelled glucose oligomers from which the retention times for the individual glycans were converted to glucose units. The data extraction was performed with an automatic processing method after which each chromatogram was manually corrected to maintain the same intervals of integration for all the samples. Finally, the chromatograms were separated into peaks in the same manner and the amount of glycans in each peak was expressed as percentage of the total integrated area.

#### Serum SOP

The blood collected for activomics analysis was mixed well within the collection tube and left to clot for at least 1 h at 4°C before centrifugation. After separation of the serum, the specimen was divided into aliquots of 500 μL using well-sealed freezing containers and immediately stored at -80°C or -20°C. For this highly sophisticated and novel analysis of enzyme activity, 2 aliquots were sent to Photeomix (S3 File). Two activomics markers were chosen to validate the sample stability before subsequent analysis for biomarkers was performed. Marker Act011has been characterized as a robust indicator of sample integrity because it measures serum proteolytic activity which is sensitive to storage conditions and potential sample mishandling. Previous cross-reference between approx. 2000 serum samples from different patients and from different centres has shown that this marker is invariant by Receiver Operator Characteristic (ROC) analysis (ROC Area Under the Curve (AUC) value 0.5 ± 0.05)except where samples have been mishandled (e.g. when left at temperatures > 22°C for several hours/days). Marker Act02 has been characterised as a sensitive indicator of different sample preparation techniques, particularly where uncontrolled lysis of blood cells can ‘contaminate’ sera with confounding cell-derived activities. Samples collected and validated, will be handled and analysed in a way that minimises freeze-thaw cycles. For each enzymatic reaction tested, 1–5 μL of serum from patients and healthy controls will be incubated with the appropriate activomics^®^ substrate under controlled conditions (time, temperature, optimised buffer conditions, etc.). Enzymatic modifications of the substrate will be monitored quantitatively by proprietary charge-based microfluidic assays for subsequent statistical analysis. The assays will be repeated for a panel of different substrates in order to provide a wide view of disease-related changes to post-translational modification activities for multivariate statistical analysis.

## Results

### DNA verification

**Fig 1:**
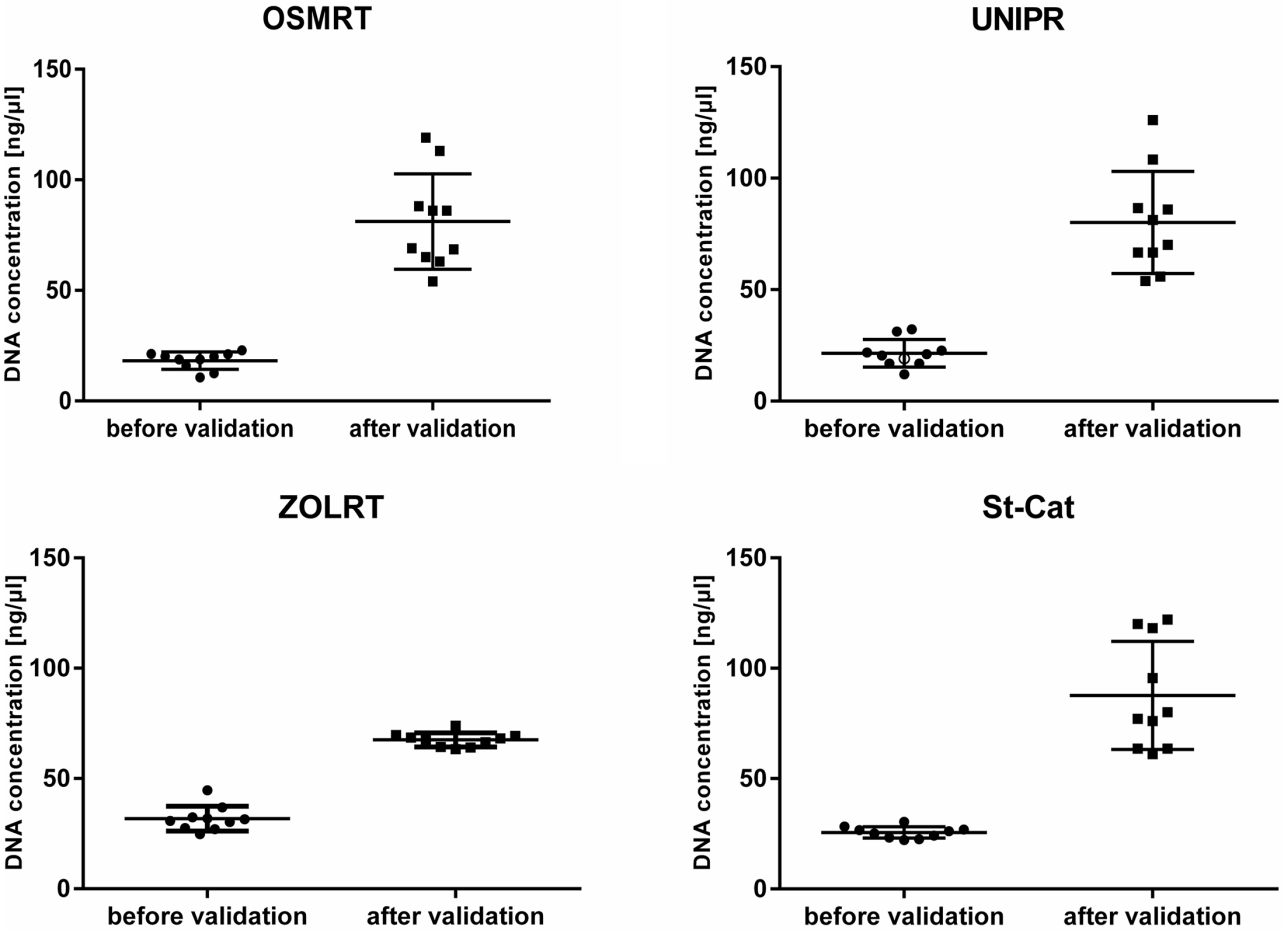
DNA concentration before and after validation. DNA concentrations (mean ± SD) of DNA test samples in duplicate. Dots indicate single DNA samples of Clinical Centres before and after SOP adjustment.

First results of quality control showed sub-par DNA quality in almost all investigated test samples. DNA samples from 4 clinical centres revealed concentrations below 50 ng/μL, mainly caused by having less starting volume of blood and differences in calibration of measuring instruments between the participating centres and the central laboratory. After increasing the amount of blood and adjusting the SOP accordingly, DNA concentrations were sufficient and reached expected values above 50 ng/μL (Fig 1). With this updated of the SOP all DNA samples successfully passed quality control and showed satisfying results concerning DNA quality and quantity. Moreover, amelogenin PCR has exactly identified the gender of samples, compared to the study databases. This process was necessary to validate the established SOP and to ensure high quality DNA samples which can then be used for further downstream analysis (e.g. GWA studies) and also to exclude any mix up of already encoded samples.

### Glycomics and plasma check

**Fig 2:**
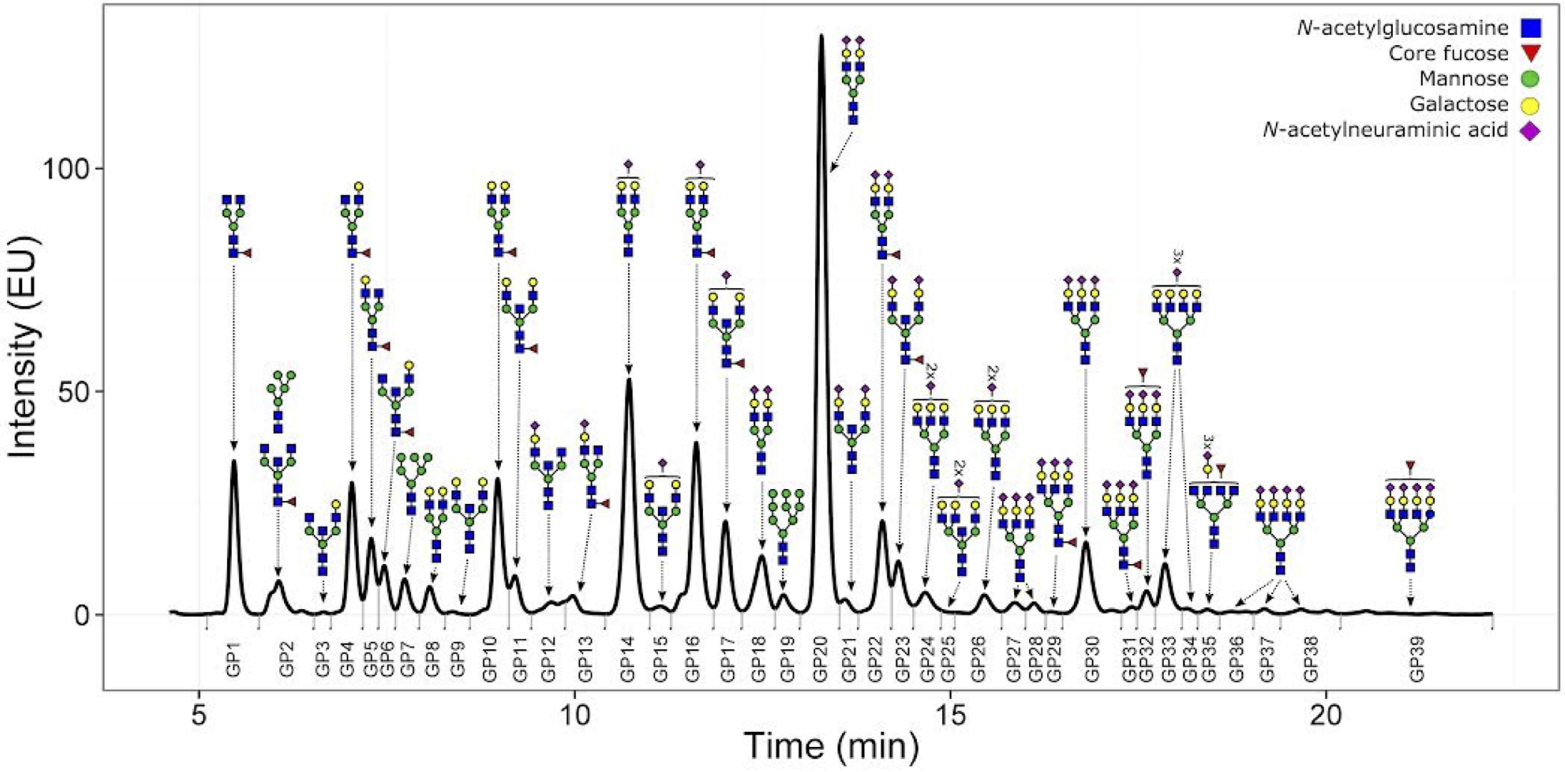
Chromatogram of 2-AB labeled N-linked glycans released from the plasma proteins and separated by HILIC-UPLC. The small vertical bars below the 0 level denote the integration area for each peak, and the major chemical structure present in each glycan group (GP1-GP39) is given above each peak. ***Data points represent medium of at least the duplicate for each samples with the P-values (-log10) for the analysis of associations between plasma glycans and LBP diagnosed at the time of blood collection for glycome analysis***.

Plasma check was performed for all 10 obtained samples. From each plasma sample 10 μL was taken and denatured with SDS and temperature. N-glycans were released with PNG-ase F and the released N-glycans were labelled with 2-AB. UPLC analysis was performed as mentioned above and the obtained chromatograms were separated into 39 glycan peaks (GP) (Fig 2). All samples passed our quality control (peak intensities, signal to noise ratio, peak separation and shape).

### Activomics and serum check

**Fig 3:**
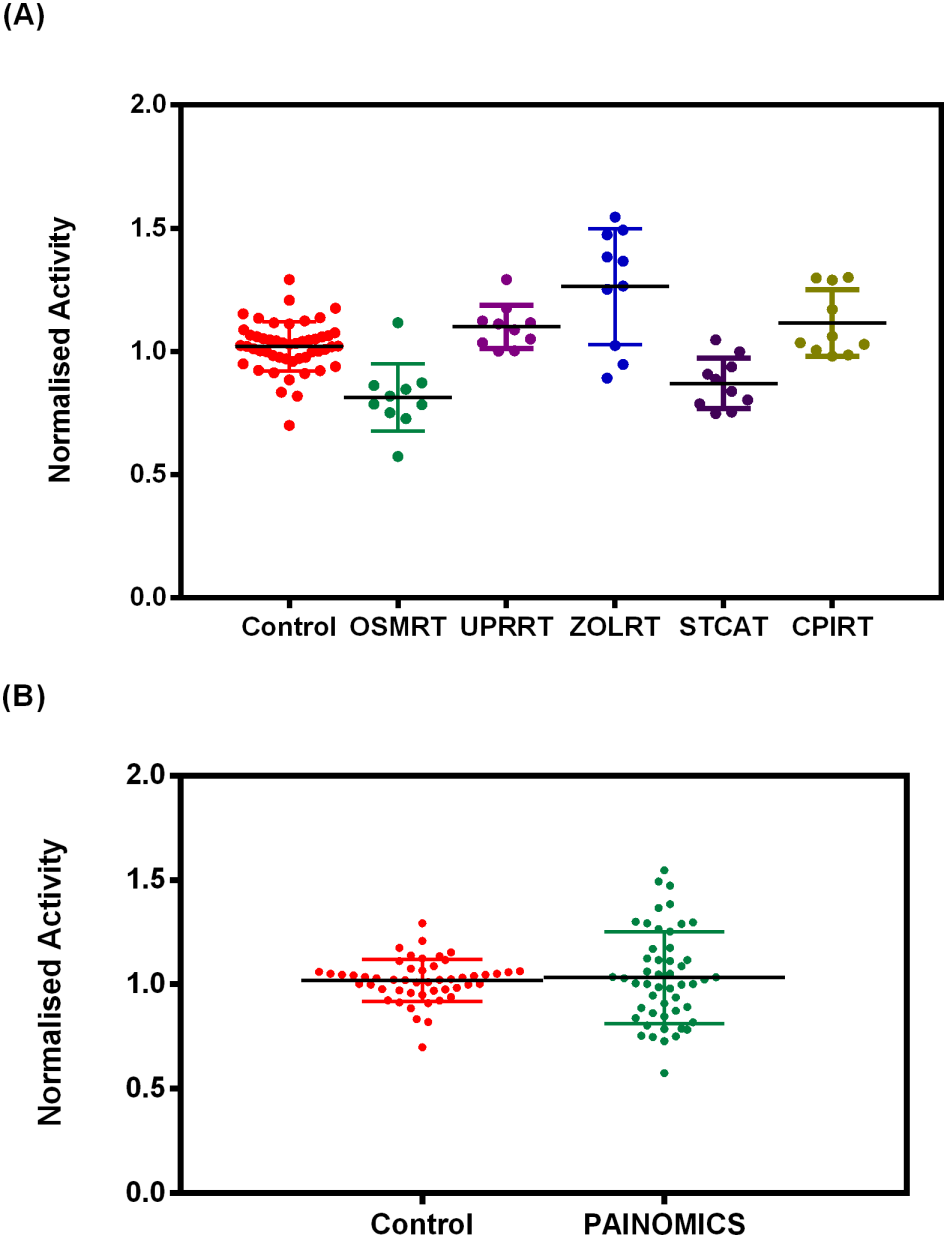
Test of sample integrity. Normalised carboxypeptidase activity for PainOmics samples analysed (*A*) by centre and (*B*) as a whole PainOmics group versus control serum samples. (Control = 1.0 ± 0.1 versus PainOmics = 1.0 ± 0.2; unpaired t test *P* = 0.6). Each data point represents an individual patient sample (average of *n* = 8 replicates).

**Fig 4:**
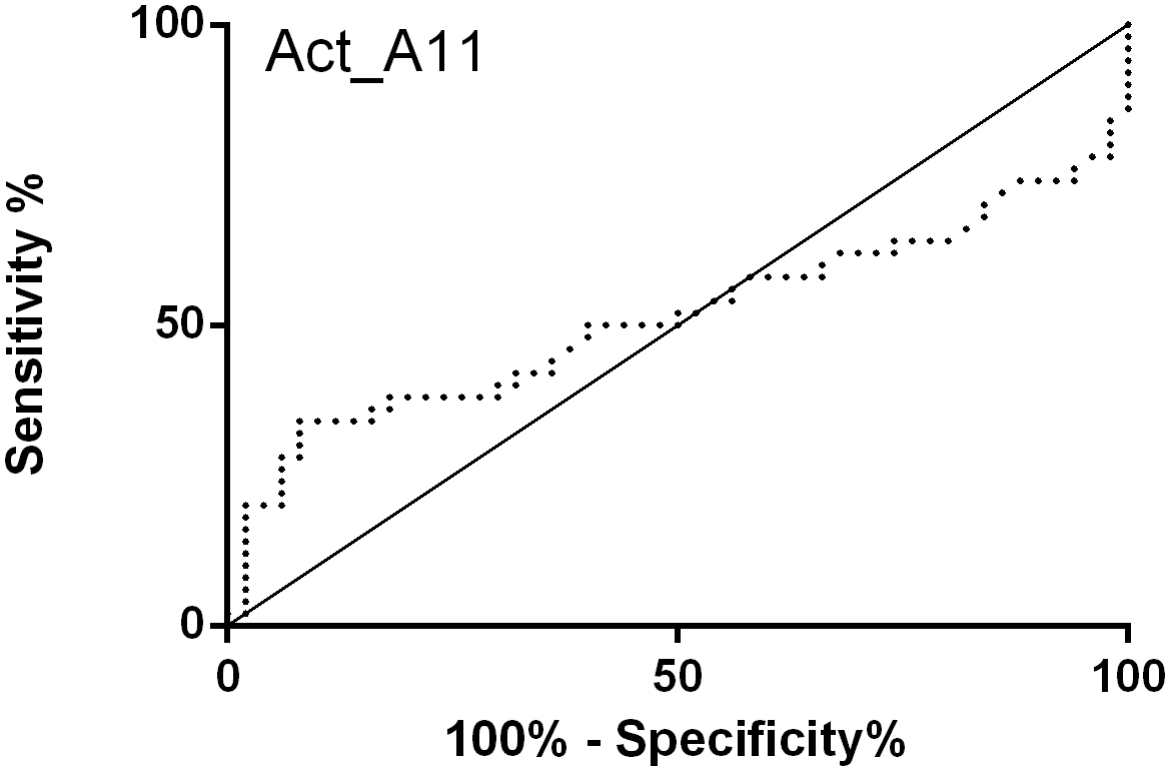
ROC analysis of normalised Act_A11 activity with PainOmics versus control serum samples. The Area Under the Curve (AUC) of 0.51 ± 0.06 indicates this marker is essentially invariant between serum controls versus PainOmics samples, thereby confirming the homogeneity of the various samples received.

The activities obtained with each set of 10 samples were compared to 10 control sera on the same screening plate and normalised to this activity to enable (Fig 3A) inter-experiment comparisons and collective analysis of the samples as a whole group (Fig 3B). This provided enough samples to perform a Receiver Operator Characteristic (ROC) analysis (*N* = 50), in order to characterise marker performance (Fig 4).

**Fig 5:**
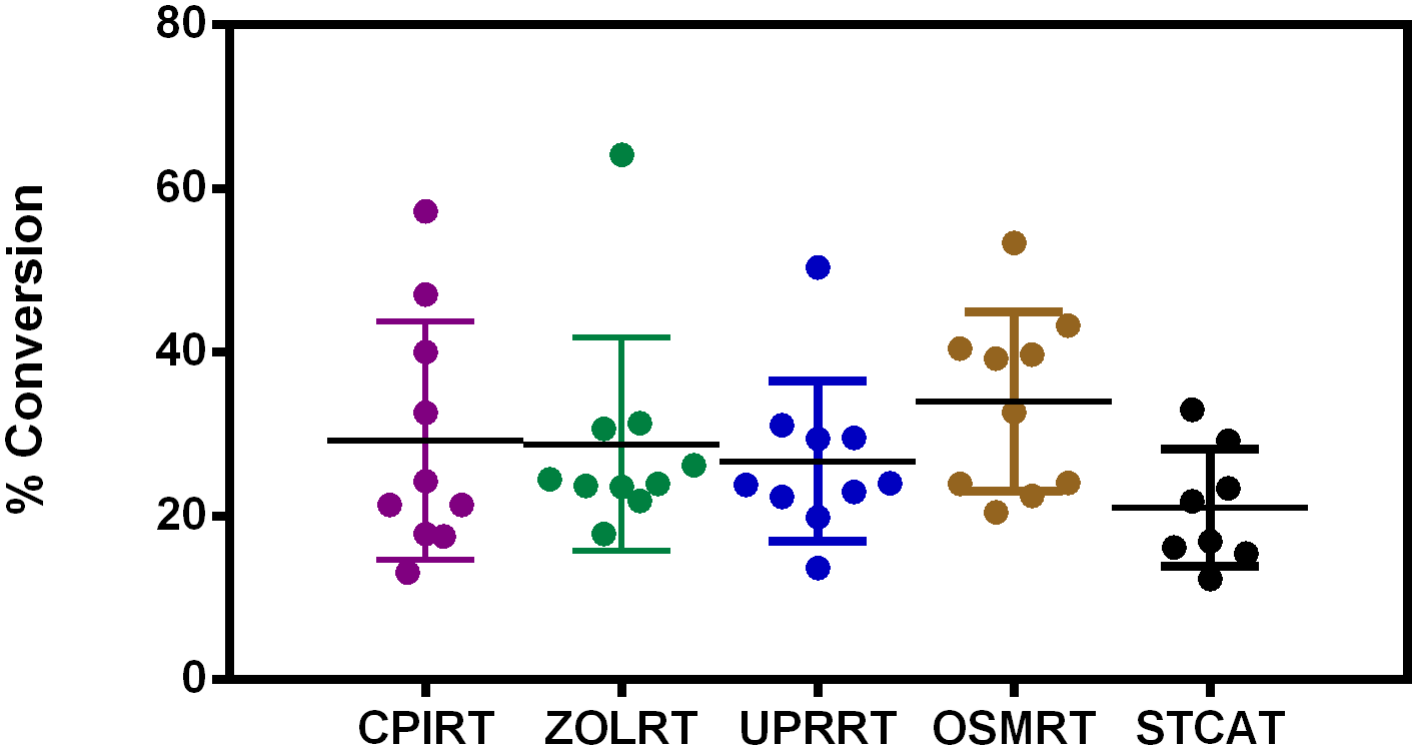
Test of sample preparation. The activity of the PainOmics samples was analysed by centre (points represent the average activity for each patient (*n* = 4). These data indicate the homogeneity of serum sample preparation for our analyses. The presence of several outliers from different centres suggest some variation exists between patient samples for this marker.

In other studies, the Act02 marker has been shown to measure erroneously high levels of activity in poorly handled samples (presumably arising from uncontrolled cell lysis), allowing accurate identification of centres using these different techniques (by ROC analysis AUC > 0.9). In the current validation study no such variation was seen between centres for the first 10 samples tested providing a further control for sample preparation, handling and transport (Fig 5).

## Discussion

Chronic Low Back Pain (CLBP) is an unresolved phenomenon sustained by complex physiological changes, which may lead to chronic syndrome with high socioeconomic costs [14]. Identification of ‘-omics’ biomarkers plays a critical role in the development of patient-specific therapies and individualized medicine, and as such offers great opportunities for fighting the current limitations of chronic pain management. The transition from acute to chronic pain is related to genetic background and post-translational modifications [15, 16], which could be investigated through genomics [17], glycomics [18] and activomics [19] approaches.

To establish consistency in our multidisciplinary and international study and the secondary integration for the correct and common preparation of biological samples among all members of the consortium, it was necessary to create comprehensive standard operating procedures (SOPs). This approach had ensured repeatability of the experiments and transferability of the data.

We have developed and validated personalised SOPs for the handling of biological samples for the management and traceability of samples from patients enrolled in a multicenter multidisciplinary -omics study, in order to guarantee reliable results, independent of the clinical centre that isolated the sample. Our results, obtained from the first 10 patients enrolled from each clinical centre, suggest that the three SOPs are suitable for the -omics analyses.

Moreover, strict adherence to these SOPs and centralized data analysis, with clinical data stored in an annotated database, it resulted in with a better reproducibility obtaining high-quality clinical endpoints correlating our genomics, glycomics and activomics data with others new -omics determinants [20]. Indeedthe study details were already published in our first manuscript[12] underling how it was important to adopt the technically sound and standardized procedures (an harmonisation of the procedures from collecting, handling, shipping to, finally, analysing of each samples)to get the best result in a large number of the patient’ samples managed in all the Centres participating in this study.

**Fig 6:**
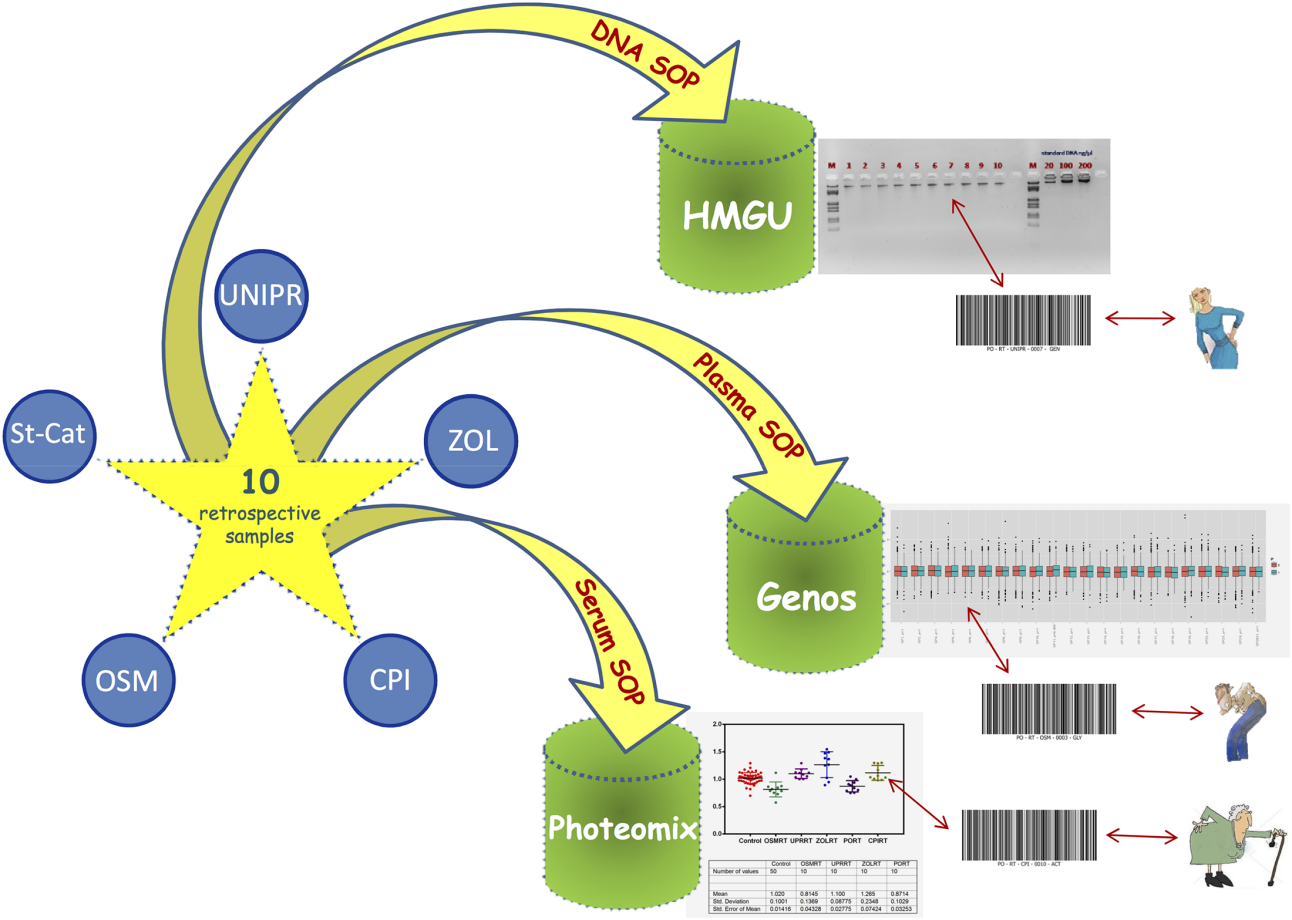
Schematic representation of the validation of personalized SOPs.

Therefore, we think that our personalized SOPs may be useful for the scientific community to manage large numbers of heterogeneous samples, having same experimental procedures and meanwhile preserving and improving respectively the individuality of the clinical and methodological aspects, usable by all without other instructions but with a simple and immediate consultation of the steps to pursue the reproducibility and accuracy of the results (Fig 6). Nevertheless, SOPs remain largely exploratory and may require a re-evaluation from the practical point of view after their implementation in the ongoing worldwide study.

Next step will be to implement even more our SOPs introducing cryotubes with a chip-readable database connected to an laboratory information management system (LIMS). In this way we can trace better all the activities performed with this sample, making it traceable and identifying the most suitable samples for subsequent analysis. All these data could be uploaded in a web-based database in order both to have a online access to all data and, eventually, to change SOPs accordingly to specific needs of the study (improving the quality of the single study).

## Supporting information

S1 File SOP-PAIN-OMICS-0002-DNA-Blood-Sampling-v2.0. (PDF)

S2 File SOP-PAIN-OMICS-0003-Plasma -Blood Sampling-v3.0. (PDF)

S3 File SOP-PAIN-OMICS-0002-Serum -Blood Sampling-v2.0. (PDF)
