# Supplementary material for: Validation of standard operating procedures in a multicenter retrospective study to identify -omics biomarkers for chronic low back pain
Source: PLoS One. 2017 May 1;12(5):e0176372. doi: 10.1371/journal.pone.0176372 (PMC5411039; doi:10.1371/journal.pone.0176372)
Supplement: S1 File — (PDF) [file pone.0176372.s001.pdf]

Project code: 602736	Date:

SOP-PAIN-OMICS-0002-DNA-Blood-Sampling-v2.0

Version Number: 2.0

Date Written: 14th April 2014

Review Date: 14th July 2014

Authors: Manuela De Gregori, Melanie Waldenberger

Position: PhD Biologist

Amended By: Manuela De Gregori, Melanie Waldenberger

Position: PhD Biologist

Date Amended: 14th July 2014

Project Code: 602736 **Operators:**

Date:

Sample codes:

PO - acronym of center - progressive number of enrolment - GEN (for GWAS study) FOR PROSPECTIVE STUDY

PO - RT - acronym of center - progressive number of enrolment - GEN (GWAS) FOR RETROSPECTIVE STUDY

PO - acronym of center - progressive number of enrolment - NEXT (for next generation sequencing study, PROSPECTIVE STUDY)

PO – OSM/UNIPR - progressive number of enrolment - Met (for methylation study) t0

PO – OSM/UNIPR - progressive number of enrolment - Met (for methylation study) t1 (after 3 months)

PO – OSM/UNIPR - progressive number of enrolment - Met (for methylation study) t1 NC* (after 3 months)

PO – OSM/UNIPR - progressive number of enrolment - Met (for methylation study) t2 (after 9 months)

NC*: not chronic pain

Project code: 602736	Date:

Objective

This document describes the procedure for obtaining patient blood for DNA analysis techniques, extracting DNA using a commercial kit and sending the DNA to laboratories

Tick and initial each box when stage is complete

Health and Safety and Personal Protective Equipment

PPE that should be worn: Clean laboratory coat, safety glasses and powder free gloves

Before starting of any blood sampling the workplace has to be well checked for cleanliness and hygiene. Between the examinations and blood collections of different study participants the surfaces of the workplace and the hands of the examiner have to be disinfected with suitable disinfectants.

Project code: 602736	Date:

Equipment, Chemicals and Consumables Required

Equipment	Asset Number	Last Calibration/Service Date
Freezer -20°C		
Freezer -80°C		
Centrifuge (necessary speed 14000 g)		
Micropipettes (10 µl,100 µl, 1 ml)		
Tourniquet		

Consumables	Item Code	BatchNo.
21G/23G butterfly needle and syringe		
Vacutainer Blood Tube (EDTA)		
Nunc cryotubes 1.8ml (or other)		
1ml Pipette tips		

Information above completed ☐

Project code: 602736	Date:

Time Line

**Procedures**	**Time**
**1 Collection of patient blood sample**	**20 min**
**2 Transfer to laboratory**	**15 min**
**3 DNA extraction**	**90 min**
**4 Freezing of Blood and DNA**	**5 min**
**Total time for procedure approximately:**	**130 min**

Method

1 Collection of patient blood sample

Per patient 2 Vacutainer tubes (EDTA tubes) are required (1 ml in each tube) (….-NEXT or …..-GEN)

Note patient details on each tube

Collect blood sample from patient using “Tourniquet + butterfly needle method”

Time sample collection finished ________________________________

Place tubes in cool box containing ice blocks and bring to lab within 6 hours. Samples should be kept cool (4°C) before the arrival to the lab

2 Transfer of samples to lab

Time of sample arrival in lab ___________________________

Maintain tubes at 4°C during the pre-processing phase if the samples are processed in the same day. Freeze tubes at -20°C if the samples are processed in a following day/days

3 DNA extraction

DNA extraction will be performed by using commercial kit which may be automated (at instance on the QIAcube), from about 200 µl of blood

The quantification of DNA will be measured by spectrophotometric method. DNA should be used at a concentration of 60 ng/µl, with a minimum 40 µl total volume; not to dilute the DNA below than 50 ng/µl.

The quality of DNA will be defined according to the 260/280 ratio (not less than 1.8) and 260/230 (between 1.8 and 2.2)

4 Aliquoting of DNA samples

Transfer approx. 2 µg of DNA in a cryotube (PO–acronym of center–progressive number of enrolment–GEN or PO–RT–acronym of center–progressive number of enrolment –GEN) and about 4 µg in another cryotube (PO–acronym of center–progressive number of enrolment–NEXT) marking all sample details on each tube.

5 Freezing of blood and DNA samples

Transfer DNA samples to -20°C or -80°C freezer. Note samples in freezer log book

Update sample storage system (books, Excel spreadsheet or LIMS software) with sample details

Time samples frozen _______________________ Date ___________________

Project code: 602736	Date:

Deviations from Procedure

Note any deviations from the procedure here, giving reasons and effects

Sign Off

PAIN-OMICS sign off by operator

Signed______________________ Date_________________

SOP sign off by supervisor

Signed______________________ Date_________________

Storage and Admin

Storage: Transfer DNA to -80°C, or -20°C freezer. Note samples in freezer log book.

Update sample storage system (books, Excel spreadsheet or LIMS software) with sample details.

Shipping of DNA

2 µg of DNA (PO–acronym of center–progressive number of enrolment–GEN or PO–RT–acronym of center–progressive number of enrolment –GEN) will be sent to Dr. Melanie Waldenberger, Helmholtz Zentrum München, Deutsches Forschungszentrum für Gesundheit und Umwelt (GmbH); Research Unit of Molecular Epidemiology, Building 34 Room 307, Ingolstädter Landstr. 1 85764 Neuherberg, Germany

Please inform Dr Melanie Waldenberger and Judith Manz (waldenberger@helmholtz-muenchen.de and judith.manz@helmholtz-muenchen.de) of the shipment.

4 µg of DNA (PO–acronym of center–progressive number of enrolment–NEXT) will be sent to Dr Zorzetto, S.C. Pneumologia, Laboratorio di Biochimica e Genetica, Padiglione Forlanini, Fondazione IRCCS Policlinico San Matteo, Viale Golgi 19, 27100 Pavia, Italy. Please inform Dr Manuela De Gregori and Michele Zorzetto (manuela.degregori@unipv.it, m.zorzetto@smatteo.pv.it) of the shipment.

1. Aim/field of application/tasks

The transport of biosamples has to be performed strictly under standardized conditions to prevent a loss of sample quality. The purpose of this Standard Operating Procedure is to harmonize the shipping conditions of biosamples

2. Responsibilities

Insert responsible person(s) here.

3. Work procedure

3.1. Description of operating procedure

Sample packing

Samples in tubes/vials

- ✓ Each tube/vial has to be clearly labelled (using a permanent marker). Use printed labels (barcodes) if possible.
- ✓ Tubes/vials should be packed in cardboard/plastic boxes, ideally in a styrofoam box (styrofoam, neopor...) with a coating thickness of at least 5 cm for adequate stability. A paperboard coated box is favored over a non-coated box. Avoid packing tubes in plastic bags. Paper toweling can be placed in the box to cushion the sample tubes/vials while transporting.

Samples in 96 well plates

- ✓ Each plates has to be clearly labelled. Use printed labels if possible.
- ✓ Plate should be firmly sealed with capmat to avoid spilling of samples.

Due to safety reasons, information concerning sender and recipient of the biosample delivery (address, contact person) are to be enclosed inside the package as well as information concerning sample type and position plan.
The paperboard or styrofoam boxes must be labelled with the required hazardous material tags (UN 1845).

Sample shipping

Shipping temperature

Ship DNA samples on dry ice. Ensure that the samples are properly packed to maintain the required temperature for the journey plus two days (see shipping days below).

The biosamples should be surrounded from all sides by a dry ice layer with a thickness of at least 5 cm. Vacuity above the dry ice layer should be filled-up with packing material or further dry ice in order to avoid a shift of the insulating bed (dry ice) during the transport. For reasons of dispersal, dry ice pellets (nuggets) are favored over dry ice blocks.

Shipping Days

Shipment of samples typically takes up to 3 days. Ideally, shipments should be sent on Monday. Avoid shipping during National holidays (always check with recipient before sending.

Shipping

Paste up the package with sender and recipient information including contact person and phone number.

Before shipping please inform the recipient on the following informations:

- ✓ Contact details
- ✓ Shipping details (shipping company, intended shipping date, shipment packaging and temperature)
- ✓ Sample details (total number of samples, complete list of samples)
- ✓ Sex of the samples (for quality checks)

After shipping, inform the recipient on waybill number for tracking of shipment.
An acknowledgement will be sent to the shipper when the samples have been received and checked.
The process is not completed till the recipient confirms the acceptance of the consignment.
