# Supplementary material for: Validation of standard operating procedures in a multicenter retrospective study to identify -omics biomarkers for chronic low back pain
Source: PLoS One. 2017 May 1;12(5):e0176372. doi: 10.1371/journal.pone.0176372 (PMC5411039; doi:10.1371/journal.pone.0176372)
Supplement: S2 File — (PDF) [file pone.0176372.s002.pdf]

SOP-PAIN-OMICS-0003-Plasma -Blood Sampling-v3.0

**Version Number:** 3.0	
**Date Written:** 14th April 2014	**Review Date:** 10th March 2015
**Author:** Mislav Novokmet, Gordan Lauc	**Position:** PhD Biologist, Professor
**Amended By:** Manuela De Gregori	**Position:** PhD Biologist
**Date Amended**: 10th March 2015	

Project Code: 602736 **Operators:**

Date:

Sample codes:
PO - acronym of center - progressive number of enrolment - GLY (for Glycomic study) t0 FOR PROSPECTIVE STUDY
PO - acronym of center - progressive number of enrolment - GLY (for Glycomic study) t1 (after 3 months) FOR PROSPECTIVE STUDY
PO - acronym of center - progressive number of enrolment - GLY (for Glycomic study) t2 (after 9 months) FOR PROSPECTIVE STUDY

PO – RT - acronym of center - progressive number of enrolment - GLY (for Glycomic study) FOR RETROSPECTIVE STUDY

PO -OSM - progressive number of enrolment - RNA (for miRNA study) t0 FOR PROSPECTIVE STUDY
PO - OSM - progressive number of enrolment - RNA (for miRNA study) t1 (after 3 months) FOR PROSPECTIVE STUDY

PO - OSM - progressive number of enrolment - RNA (for miRNA study) t2 (after 9 months) FOR PROSPECTIVE STUDY

Objective

This document describes the procedure for obtaining patient blood for plasma analysis techniques, extracting plasma and sending the plasma to laboratories.

Tick and initial each box when stage is complete.

Health and Safety and Personal Protective Equipment

PPE that should be worn: Clean laboratory coat, safety glasses and powder free gloves ☐

Before starting of any blood sampling the workplace has to be well checked for cleanliness and hygiene. Between the examinations and blood collections of different study participants the surfaces of the workplace and the hands of the examiner have to be disinfected with suitable disinfectants.

Project code: 602736	Date:

Equipment, Chemicals and Consumables Required

Equipment	Asset Number	Last Calibration/Service Date
Centrifuge		
Freezer -80°C/-20°C		
1ml pipette		
Tourniquet		

Consumables	Item Code	Batch No.
21G/23G butterfly needle and syringe		
Vacutainer Blood Tube (EDTA)		
Nunc cryotubes 1.8mL (or other)		
1ml Pipette tips		
2ml Eppendorf tubes		

Information above completed ☐

Project code: 602736	Date:

Time Line for Plasma Procedure

Procedure		Time
**1**	**Collection of 1 patient blood sample**	**20 min**
**2**	**Transfer to laboratory**	**15 min**
**3**	**Leave the tube resting at the room temperature**	**60 min**
**4**	**Centrifugation of plasma**	**10 min prep 10 min actual centrifugation**
**5**	**Transfer of plasma**	**10 min**
**6**	**Freezing of plasma**	**5 min**

Total time for procedure approximately: **130 minutes**

Method

1 Collection of blood samples

Per patient 1 Vacutainer tube (EDTA tube) is required (2 ml).
Note patient details on tube.
Collect blood sample from patient using "Tourniquet + butterfly needle method".

Time sample collection finished ____________________________

Place tubes in cool box containing ice blocks and bring to lab within 6 hours. Samples should be kept cool (4°C) before the arrival to the lab.

2 Transfer of sample to lab

Time of sample arrival in lab ____________________________

.
SAMPLES MUST BE PROCESSED ON THE SAME DAY UPON ARRIVAL AT THE LAB!

3 First centrifugation of plasma

Leave the tube resting at the room temperature for an hour.
Place the tube in a centrifuge to centrifuge it.
Ensure tube is balanced with equivalent water containing tube.
Set centrifuge to spin for 10 minutes at 1620 g.

4 Transfer of plasma

Transfer plasma to a 2 mL eppendorf tube. Mark patient and sample details.
Do not disturb plasma/buffy coat interface.

5 Second centrifugation of plasma

Place the eppendorf in a centrifuge.
Ensure tubes are properly balanced.
Set centrifuge to spin for 10 minutes at 2700 g.

6 Transfer of plasma

Transfer 1 ml of plasma to a 2 mL cryotube, mark all sample details.

7 Freezing of plasma samples

Transfer processed plasma to -80°C, or -20°C freezer. Note samples in freezer log book.

Time samples frozen ______________________ Date _________________

Deviations from Procedure

Note any deviations from the procedure here, giving reasons and effects

Sign Off

PAIN-OMICS sign off by operator   Supervisor

Signed__________________ Date______________   Signed__________________ Date______________

Project code: 602736	Date:

Storage and Admin

Storage: processed plasma to -80°C, or -20°C freezer. Note samples in freezer log book.
Update sample storage system (books, Excel spreadsheet or LIMS software) with sample details.

Sign Off

PAIN-OMICS sign off by operator

Signed___________________ Date_______________

SOP sign off by supervisor

Signed___________________ Date_______________

Shipping of plasma samples

About 1 ml of plasma will be sent into cryotube to Professor Gordan, GENOS, Hondlova 2/11, 10000 Zagreb, Croatia. Please inform prof Gordan Lauc (glauc@genos.hr) of the shipment.

Aim/field of application/tasks

The transport of biosamples has to be performed strictly under standardized conditions to prevent a loss of sample quality. The purpose of this Standard Operating Procedure is to harmonize the shipping conditions of biosamples.

Project code: 602736	Date:

1. Responsibilities

Insert responsible person(s) here.

2. Work procedure

2.1. Description of operating procedure

Sample packing

Samples in tubes/vials

- ✓ Each tube/vial has to be clearly labeled (using a permanent marker). Use printed labels (barcodes) if possible.
- ✓ Tubes/vials should be packed in cardboard/plastic boxes, ideally in a styrofoam box (styrofoam, neopor...) with a coating thickness of at least 5 cm for adequate stability. A paperboard coated box is favored over a non-coated box. Avoid packing tubes in plastic bags. Paper toweling can be placed in the box to cushion the sample tubes/vials while transporting.

Samples in 96 well plates

- ✓ Each plate has to be clearly labeled. Use printed labels if possible.
- ✓ Plate should be firmly sealed with capmat to avoid spilling of samples.

Due to safety reasons, information concerning sender and recipient of the biosample delivery (address, contact person) are to be enclosed inside the package as well as information concerning sample type and position plan.
The paperboard or styrofoam boxes must be labeled with the required hazardous material tags (UN 1845).

Sample shipping

Shipping temperature

Ship plasma samples on dry ice. Ensure that the samples are properly packed to maintain the required temperature for the journey plus two days (see shipping days below).

The biosamples should be surrounded from all sides by a dry ice layer with a thickness of at least 5 cm. Vacuity above the dry ice layer should be filled-up with packing material or further dry ice in order to avoid a shift of the insulating bed (dry ice) during the transport. For reasons of dispersal, dry ice pellets (nuggets) are favored over dry ice blocks.

Shipping Days

Shipment of samples typically takes up to 3 days. Ideally, shipments should be sent on Monday. Avoid shipping during National holidays (always check with recipient before sending).

Shipping

Paste up the package with sender and recipient information including contact person and phone number.
Before shipping please inform the recipient on the following informations:

- ✓ Contact details
- ✓ Shipping details (shipping company, intended shipping date, shipment packaging and temperature)
- ✓ Sample details (total number of samples, complete list of samples)
- ✓ Sex of the samples (for quality checks)

After shipping, inform the recipient on waybill number for tracking of shipment.
An acknowledgement will be sent to the shipper when the samples have been received and checked.
The process is not completed till the recipient confirms the acceptance of the consignment.
