# Supplementary material for: Validation of standard operating procedures in a multicenter retrospective study to identify -omics biomarkers for chronic low back pain
Source: PLoS One. 2017 May 1;12(5):e0176372. doi: 10.1371/journal.pone.0176372 (PMC5411039; doi:10.1371/journal.pone.0176372)
Supplement: S3 File — (PDF) [file pone.0176372.s003.pdf]

SOP-PAIN-OMICS-0002-Serum -Blood Sampling-v2.0

**Version Number:** 2.0	
**Date Written:** 14th April 2014	**Review Date:** 14th July 2014
**Author: Iain Pemberton, Jane MacDougall**	**Position: Professors**
**Amended By: Iain Pemberton, Jane MacDougall**	**Position: Professors**
**Date Amended**: **14th July 2014**	

**Project Code: 602736**	**Operators:**

Date:

Sample codes:
PO - acronym of center - progressive number of enrolment - ACT (for Activomic study) t0 FOR PROSPECTIVE STUDY
PO - acronym of center - progressive number of enrolment - ACT (for Activomic study) t1NC* (after 3 months) FOR PROSPECTIVE STUDY
PO - acronym of center - progressive number of enrolment - ACT (for Activomic study) t1 (after 3 months) FOR PROSPECTIVE STUDY
PO - acronym of center - progressive number of enrolment - ACT (for Activomic study) t2 (after 9 months) FOR PROSPECTIVE STUDY

PO – RT - acronym of center - progressive number of enrolment - ACT (for Activomic study) FOR RETROSPECTIVE STUDY

NC*: patients without chronic pain

Objective

This document describes the procedure for obtaining patient blood for Activomics analysis techniques.

Tick and initial each box when stage is complete.

Health and Safety and Personal Protective Equipment

PPE that should be worn: Clean laboratory coat, safety glasses and powder free gloves.

Before starting of any blood sampling the workplace has to be well checked for cleanliness and hygiene. Between the examinations and blood collections of different study participants the surfaces of the workplace and the hands of the examiner have to be disinfected with suitable disinfectants.

Project code: 602736	Date:

Equipment, Chemicals and Consumables Required

Equipment	Asset Number	Last Calibration/Service Date
Centrifuge		
Freezer -80°C/-20°C		
1ml pipette		
Tourniquet		

Consumables	Item Code	Batch No.
21G/23G butterfly needle and syringe		
Serum tube with clot activator plus gel	Greiner 455092	
Nunc cryotubes 1.8ml (or other)		
1ml Pipette tips		

Information above completed ☐

Time Line for Serum Procedure

Procedure		Time
**1**	**Collection of 1 patient blood sample**	**20 min**
**2**	**Transfer to laboratory**	**15 min**
**3**	**Clotting of sample**	**60 min minimum**
**3**	**Centrifugation of samples**	**15 min actual centrifugation**
**5**	**Transfer of serum**	**10 min per sample**
**6**	**Freezing of serum**	**5 min**
**Total time for procedure approximately:**		**90 min**

Method

1 Collection of blood samples

Per patient 1 serum tube is required (2 ml)
Note patient details on each tube
Collect blood sample from patient using “Tourniquet + butterfly needle method”

Time sample collection finished ______________________________

Place tubes in cool box containing ice blocks and bring to lab within 6 hours. Samples should be kept cool (4°C) before the arrival to the lab.

2 Transfer of samples to lab

Time of sample arrival in lab ______________________________

SAMPLES MUST BE PROCESSED ON THE SAME DAY UPON ARRIVAL AT THE LAB!

3 Clotting of samples

Leave the tubes at 4°C for 60 minutes to allow completion of blood clotting.

4 Centrifugation of sample

Place the tube of clotted blood in a centrifuge
Ensure tubes are properly balanced
Set centrifuge to spin for 15±5 minutes at 2500±500 g. at 4°C
Time and date of centrifugation
Time: ______________________ **Date:** __________________________

5 Aliquoting of serum samples

Transfer approx. 0.5 ml serum ~2 cryotubes, mark all sample details on each tube.

6 Freezing of serum samples

Transfer processed serum to -80°C, or -20°C freezer. Note samples in freezer log book.

Time samples frozen _________________________ Date ___________________

Deviations from Procedure

Note any deviations from the procedure here, giving reasons and effects

Sign Off

PAIN-OMICS sign off by operator Supervisor

Signed____________________ Date________________ Signed____________________ Date________________

Storage and Admin

Storage: processed serum to -80°C, or -20°C freezer. Note samples in freezer log book.
Update sample storage system (books, Excel spreadsheet or LIMS software) with sample details.

Sign Off

PAIN-OMICS sign off by operator

Signed___________________ Date_______________

SOP sign off by supervisor

Signed___________________ Date_______________

Shipping of serum samples

About 1 ml of serum into the cryotubes will be sent to Professor Pemberton, PHOTEOMIX, Rez de Chaussée, 32-34 Rue Carnot, 93160 Noisy Le Grand, France.
Please inform Prof Iain Pemberton and Jane Mac Dougall (ipemberton@photeomix.com, jmacdougall@photeomix.com) of the shipment.

Aim/field of application/tasks

The transport of biosamples has to be performed strictly under standardized conditions to prevent a loss of sample quality. The purpose of this Standard Operating Procedure is to harmonize the shipping conditions of biosamples

1. Responsibilities

Insert responsible person(s) here.

2. Work procedure

2.1. Description of operating procedure

Sample packing

Samples in tubes/vials

- ✓ Each tube/vial has to be clearly labelled (using a permanent marker). Use printed labels (barcodes) if possible.
- ✓ Tubes/vials should be packed in cardboard/plastic boxes, ideally in a styrofoam box (styrofoam, neopor...) with a coating thickness of at least 5 cm for adequate stability. A paperboard coated box is favored over a non-coated box. Avoid packing tubes in plastic bags. Paper towelling can be placed in the box to cushion the sample tubes/vials while transporting.

Samples in 96 well plates

- ✓ Each plate has to be clearly labelled. Use printed labels if possible.
- ✓ Plate should be firmly sealed with capmat to avoid spilling of samples.

Due to safety reasons, information concerning sender and recipient of the biosample delivery (address, contact person) are to be enclosed inside the package as well as information concerning sample type and position plan.

The paperboard or styrofoam boxes must be labeled with the required hazardous material tags (UN 1845).

Sample shipping

Shipping temperature

Ship serum samples on dry ice. Ensure that the samples are properly packed to maintain the required temperature for the journey plus two days (see shipping days below).

The biosamples should be surrounded from all sides by a dry ice layer with a thickness of at least 5 cm. Vacuity above the dry ice layer should be filled-up with packing material or further dry ice in order to avoid a shift of the insulating bed (dry ice) during the transport. For reasons of dispersal, dry ice pellets (nuggets) are favoured over dry ice blocks. Biological samples that degrade when frozen should be shipped on blue ice (4 °C).

Shipping Days

Shipment of samples typically takes up to 3 days. Ideally, shipments should be sent on Monday. Avoid shipping during National holidays (always check with recipient before sending).

Shipping

Paste up the package with sender and recipient information including contact person and phone number.

Project code: 602736	Date:

Before shipping please inform the recipient of the following information:

- ✓ Contact details
- ✓ Shipping details (shipping company, intended shipping date, shipment packaging and temperature)
- ✓ Sample details (total number of samples, complete list of samples)
- ✓ Sex of the samples (for quality checks)

After shipping, inform the recipient on waybill number for tracking of shipment.

An acknowledgement will be sent to the shipper when the samples have been received and checked.

The process is not completed till the recipient confirms the acceptance of the consignment.
